# Bioaccumulation and Distribution of Indospicine and Its Foregut Metabolites in Camels Fed *Indigofera spicata*

**DOI:** 10.3390/toxins11030169

**Published:** 2019-03-19

**Authors:** Gabriele Netzel, Eddie T. T. Tan, Mukan Yin, Cindy Giles, Ken W. L. Yong, Rafat Al Jassim, Mary T. Fletcher

**Affiliations:** 1 Queensland Alliance for Agriculture and Food Innovation (QAAFI), The University of Queensland, Health and Food Sciences Precinct, Coopers Plains, QLD 4108, Australia; g.netzel@uq.edu.au (G.N.); eddietan@ns.uitm.edu.my (E.T.T.T.); mukan.yin@uq.net.au (M.Y.); r.aljassim@uq.edu.au (R.A.J.); 2 Alliance of Research and Innovation for Food (ARIF), Faculty of Applied Sciences, Universiti Teknologi MARA, Cawangan Negeri Sembilan, Kuala Pilah Campus, Negeri Sembilan 72000, Malaysia; 3 Department of Agriculture and Fisheries, Health and Food Sciences Precinct, Coopers Plains, QLD 4108, Australia; cindy.giles@daf.qld.gov.au (C.G.); ken.yong@daf.qld.gov.au (K.W.L.Y.)

**Keywords:** indospicine, 2-aminopimelamic acid, 2-aminopimelic acid, in vivo, foregut metabolites, camel, food safety

## Abstract

In vitro experiments have demonstrated that camel foregut-fluid has the capacity to metabolize indospicine, a natural toxin which causes hepatotoxicosis, but such metabolism is in competition with absorption and outflow of indospicine from the different segments of the digestive system. Six young camels were fed *Indigofera spicata* (337 µg indospicine/kg BW/day) for 32 days, at which time three camels were euthanized. The remaining camels were monitored for a further 100 days after cessation of this indospicine diet. In a retrospective investigation, relative levels of indospicine foregut-metabolism products were examined by UHPLC-MS/MS in plasma, collected during both accumulation and depletion stages of this experiment. The metabolite 2-aminopimelamic acid could be detected at low levels in almost all plasma samples, whereas 2-aminopimelic acid could not be detected. In the euthanized camels, 2-aminopimelamic acid could be found in all tissues except muscle, whereas 2-aminopimelic acid was only found in the kidney, pancreas, and liver tissues. The clearance rate for these metabolites was considerably greater than for indospicine, which was still present in plasma of the remaining camels 100 days after cessation of *Indigofera* consumption.

## 1. Introduction

There are more than 60 *Indigofera* species distributed throughout the arid and semiarid regions of Australia [1,2,3,4]. *Indigofera* spp. are leguminous shrubs and herbs which are high in protein, as well as highly palatable for animals. These plants are considered a nutritious animal fodder, however, some species contain indospicine, a non-proteinogenic arginine analogue which causes hepatotoxicosis in sheep, cows, rabbits, and dogs [5,6,7,8,9]. The introduced species, *Indigofera spicata*, has been found to contain high levels of this amino acid. Since there is no known mammalian enzyme which is capable of degrading indospicine [10], it can be toxic to simple-stomached animals. However, ruminants seem to be less susceptible to certain toxins due to the ability of rumen microflora to degrade some of those foodborne toxins. Feral camels in Australia seem to cope better in detoxification of plant toxins from their fodder, compared to domesticated animals [11].

Indospicine is a water-soluble free amino acid and rumen degradation processes compete with presumed rapid passage through the fermentation compartments of the digestive tract. This amino acid has been detected in meat, which indicates that at least a portion of indospicine can by-pass the fermentation processes of the foregut and be absorbed as is [12]. A number of dogs have died of secondary hepatotoxicosis after consuming indospicine-contaminated horse meat [7] and, more recently, camel meat [5], and this has raised food safety concerns. In a recent study, we have shown that indospicine accumulated in camel meat during a feeding trial in which six camels were fed a diet containing *Indigofera spicata* for 32 days, and that indospicine can be detected in plasma as long as three months after removing *Indigofera* from the diet [13]. 

**Figure 1:**
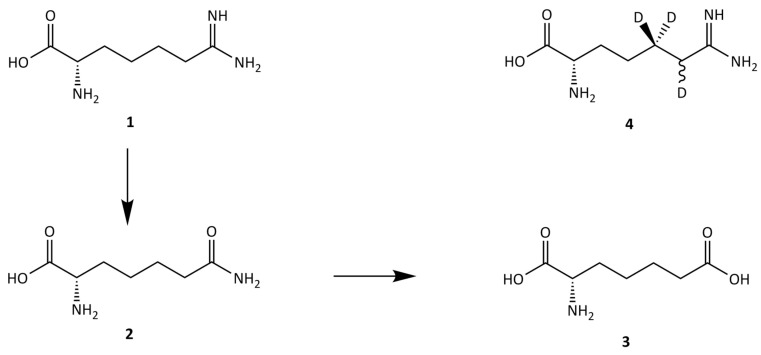
Chemical structures of indospicine (**1**) and its metabolites 2-aminopimelamic acid (**2**) and 2-aminopimelic acid (**3**), together with D_3_-l-indospicine (**4**) which is used as an internal standard in LC-MS/MS analysis.

We also reported previously that microflora of both the bovine rumen and camel foregut fluids have the ability to degrade indospicine in vitro within an incubation period of 48 h [14]. However, the in vitro degradability of indospicine is indicative of the potential degradability, and not the actual degradability, that occurs in the animal system. Factors including the microbial community, residence time of the solid fraction of digesta, and outflow rate of the fluid phase all play an important role. Camels are known to retain low quality fibre diets longer in the foregut compared with ruminant animals. Retention time is always shorter when the diet is of higher quality, which should be the case with lush early season pasture containing *Indigofera spicata* at the start of the wet season. Shift to such diet increases the outflow rate and allows more indospicine to enter the intestines where it then gets absorbed. Indospicine has been shown to be chemically stable and resistant to both acidic and base conditions [15,16]. Since the camel foregut fluid is only mildly acidic, it is most likely that rumen bacteria are responsible for the observed metabolism of indospicine (**1**) into its degradation product 2-aminopimelamic acid (**2**) and, further, to 2-aminopimelic acid (**3**) (Figure 1) [14,17].

Although we could show previously that indospicine accumulated as a free amino acid in various animal tissues in vivo [13], it has also been demonstrated that indospicine can be metabolized in vitro by foregut microbiota [14]. These two processes of removal (outflow and absorption) and metabolism could be considered to operate in competition, and there is nothing known about the extent of in vivo metabolism of indospicine and whether the metabolites are also transported and accumulated in tissues. Hence, in the present study, we investigated the bioaccumulation and distribution, as well as the excretion, of the indospicine foregut metabolites, 2-aminopimelamic acid and 2-aminopimelic acid, in camels fed *Indigofera* plant material for 32 days.

## 2. Results and Discussion

### 2.1. Indospicine and Foregut Metabolites in Tissue Samples

It has previously been established that indospicine accumulates in muscle and other tissues of cattle [18] and camels [13] fed *Indigofera* plant material, however nothing is known about the fate of the indospicine metabolites, 2-aminopimelamic acid and 2-aminopimelic acid. In this study we have measured both indospicine and the two metabolite concentrations in tissues acquired during the previous camel feeding trial, where six young camels (camels 1–6) were fed *Indigofera spicata* for 32 days until indospicine levels in plasma plateaued. At this point, three animals (camels 1–3) were euthanized and the remaining camels (camels 4–6) fed an *Indigofera*-free diet for a further 100 days whilst monitoring decline in indospicine plasma levels [13]. The inclusion rate of *Indigofera spicata* was designed to provide 337 µg of indospicine per kg BW per day.

**Figure 2:**
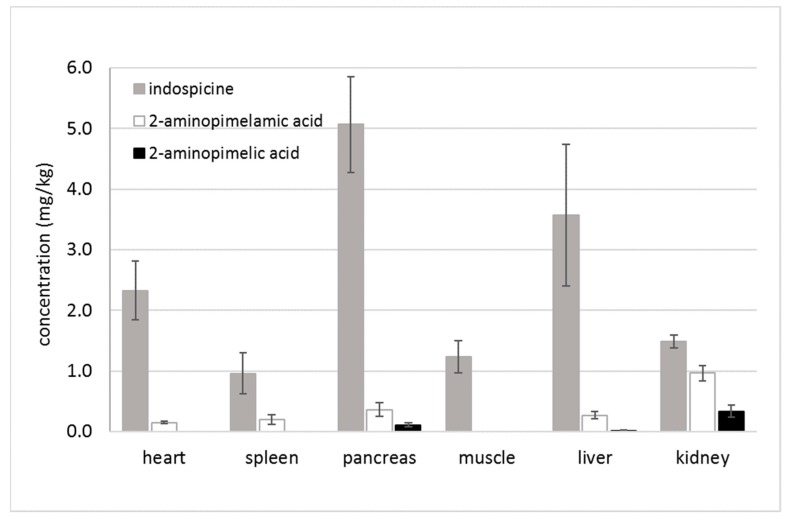
Mean concentrations (mg/kg FW) of indospicine, 2-aminopimelamic acid, and 2-aminopimelic acid for camels 1–3 necropsy tissues (heart, spleen, pancreas, muscle, liver and kidney) after 32-day feeding period.

In accord with the previous analysis, the highest concentration of indospicine in necropsied camels (camels 1–3) was found, in this study, in the pancreas (5.06 ± 0.79 mg/kg FW), followed by the liver (3.57 ± 1.17 mg/kg FW), heart (2.32 ± 0.48 mg/kg FW), kidney (1.48 ± 0.11 mg/kg FW), muscle (1.24 ± 0.26 mg/kg FW), and spleen (0.96 ± 0.34 mg/kg FW) (Figure 2). However, if we look at the deamino metabolites, the highest concentration of the intermediate metabolite 2-aminopimelamic acid was found in the kidney (0.96 ± 0.12 mg/kg FW), followed by the pancreas (0.36 ± 0.11 mg/kg FW), liver (0.27 ± 0.06 mg/kg FW), spleen (0.20 ± 0.08 mg/kg FW), and heart (0.15 ± 0.02 mg/kg FW). Neither 2-aminopimelamic acid nor 2-aminopimelic acid could be detected in the muscle tissue. Only low concentrations of the second metabolite 2-aminopimelic acid could be found in kidney (0.34 ± 0.10 mg/kg FW), pancreas (0.11 ± 0.03 mg/kg FW), and liver (0.01 ± 0.02 mg/kg FW).

The kidney, spleen, heart, liver, and pancreas are all organs where arginine metabolism plays an important role in metabolic pathways. Indospicine has, for example, been shown to be a competitive inhibitor of hepatic arginase [19], but arginase does not occur only in the liver. Arginase has two isoforms: arginase I, a cytoplasmic enzyme, which is highly expressed in the liver, and arginase II, a mitochondrial enzyme more widely distributed in extrahepatic tissues, which is expressed primarily in the kidney [20]. Arginase is a key enzyme of the urea cycle and is also present in other tissues such as the spleen, heart, kidney, and pancreas [20,21,22]. Arginase activity is reportedly blocked through the competitive binding of indospicine [19], which is consistent with the observed elevated presence of indospicine in these same tissues (Figure 2). It is worth noting that 2-aminopimelamic acid is an analogue of citrulline, in the same manner that indospicine is an analogue of arginine. We can hypothesize then that the high concentration of 2-aminopimelamic acid found in the kidney, spleen, heart, liver, and pancreas could likewise be due to interference in citrulline metabolism within the urea cycle, potentially blocking the enzyme argininosuccinate synthetase which utilises citrulline in the synthesis of argininosuccinic acid [22]. The kidney is noted to have a significant capacity for citrulline metabolism [22], and the highest level of accumulation of the citrulline analogue 2-aminopimelamic acid may reflect interference in this metabolism. Such interference could then be consistent with the dramatically elevated citrulline levels reported in indospicine treated rats [23].

### 2.2. Indospicine and Foregut Metabolites in Plasma

**Figure 3:**
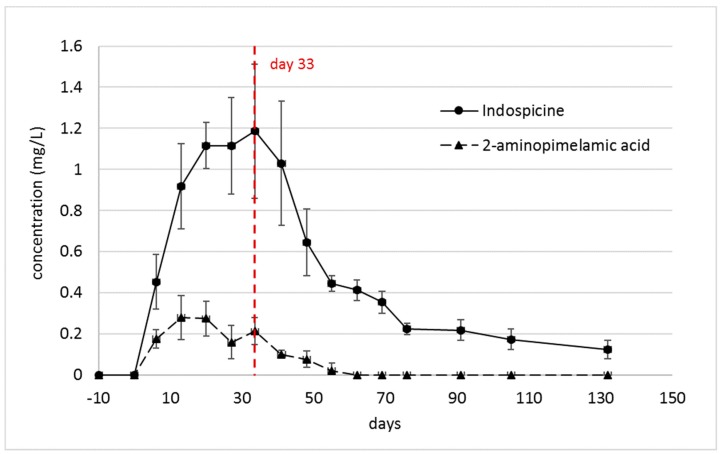
Comparison of indospicine and 2-aminopimelic acid concentrations in plasma (mean ± SD) during the first 32 days (*n* = 6) of the treatment and after cessation of *Indigofera spicata* feeding (*n* = 3). Camels 1–3 were autopsied at day 33.

The concentration of indospicine [13], as well as that of 2-aminopimelamic acid, was observed to rapidly increase in plasma of camels 1–6 in the first 13–20 days after commencement of the feeding trial (Figure 3). The indospicine concentration reached a plateau phase before it decreased slowly after ceasing the *Indigofera* uptake. The concentration of the 2-aminopimelamic acid similarly rose in the first 13 days, plateauing for about two weeks, before it slightly decreased and rose to a second maximum at 32 days, the point at which the *Indigofera* intake was stopped. In the depletion phase, when the remaining camels (camels 4–6) were fed an *Indigofera*-free diet, the 2-aminopimelamic acid concentration followed the indospicine concentration in a slow decrease until no 2-aminopimelamic acid was detectable at 62 days (30 days after cessation of *Indigofera* consumption).

**Figure 4:**
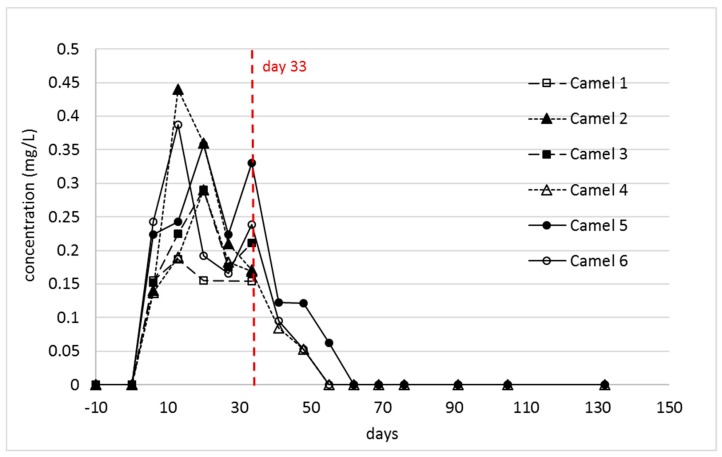
Individual 2-aminopimelamic acid concentrations of plasma during the first 32 days (*n* = 6) of the treatment and after cessation of *Indigofera spicata* feeding (*n* = 3). Camels 1–3 were autopsied at day 33.

The plasma concentrations of the 2-aminopimelamic acid were much lower than those of indospicine. As already described, there was a considerable variation in the indospicine plasma levels between the individual camels [13], but the individual indospicine plasma curves all followed a similar pattern. By comparison, the pattern of the 2-aminopimelamic acid plasma content proved to be quite variable between the camels (Figure 4). All camels showed a rapid increase within the first 13 or 20 days, to levels between 0.19–0.44 mg/L, with considerable variation between individuals. During the depletion phase, the plasma levels of the three camels (camels 4–6) followed a steady decrease until day 55 or 60, with 2-aminopimelamic acid being eliminated from the system much quicker than indospicine, which is still detected in plasma at 130 days—100 days after cessation of *Indigofera* intake (Figure 3). 

The rapid increase in plasma levels of the 2-aminopimelamic acid during the feeding phase followed the rapid increase of indospicine, which can be metabolized in the foregut to 2-aminopimelamic acid and, further, to 2-aminopimelic acid. This second metabolite 2-aminopimelic acid was not detectable in any of the plasma samples. Since no ‘fresh’ indospicine was uptaken after cessation of the *Indigofera* feeding, it is apparent that the metabolites were eliminated much faster from the camel’s system, compared to the indospicine. The lengthier persistence of indospicine residues is attributed to the slow release of this arginine analogue from organ tissue, such as the pancreas, where it was accumulated. 

## 3. Conclusions

These results demonstrate that foregut metabolism of indospicine does occur in vivo, and that the two metabolites, 2-aminopimelamic acid and 2-aminopimelic acid, are both absorbed and bioaccumulate in a range of tissues. After consumption of *Indigofera* plants, the intermediate metabolite 2-aminopimelamic acid was present in all camel tissues except muscle, with highest levels measured in the kidney, pancreas, and liver. Lower levels of 2-aminopimelic acid were also found in these same three tissues. Cytotoxicity studies conducted by Sultan et al. [24] demonstrated that the second metabolite, 2-aminopimelic acid, is less toxic than indospicine. However, nothing to date is known about the toxicity of its precursor, 2-aminopimelamic acid, so there is a possibility that this intermediate metabolite, which is an analogue of citrulline, also contributes to the effects of *Indigofera* poisoning. The clearance of this metabolite from plasma occurs faster compared to indospicine (after cessation of *Indigofera* intake), therefore the presence of 2-aminopimelamic acid in plasma could potentially be used as an indicator of whether the indospicine contamination is recent, within the past month, or longer. The actual route of excretion of indospicine (or its metabolites) has not been investigated, and it is recommended that further trials be conducted to determine levels of indospicine and its metabolites in both milk and urine of animals consuming *Indigofera.*

## 4. Materials and Methods 

### 4.1. Standards and Reagents 

Indospicine (**1**) and 2-aminopimelamic acid (**2**) (>99% pure), both external standards, as well as D_3_-l-indospicine (**4**) (>99% pure) as internal standard, were synthesized and provided by Prof. James De Voss and Dr. Robert Lang, The University of Queensland [16,25]. Another external standard, 2-aminopimelic acid (**3**) (>99% pure), and heptafluorobutyric acid (HFBA), ion chromatography grade, were purchased from Sigma Aldrich (Castle Hill, NSW, Australia). External (0.005–2 mg/L) and internal (1 mg/L) standard solutions were prepared in 0.1% HFBA in Milli-Q water.

### 4.2. Tissue Collection

Tissue samples of a previous animal trial [13] (which had been stored frozen at −20 °C) were re-analyzed to focus on the accumulation, distribution, and persistence of foregut deamino metabolites of indospicine during and after cessation of the feeding period. 

Briefly, in this study six camels (2–4 years) with a weight between 270–387 kg were fed with dried and chaffed *Indigofera spicata* to deliver 337 µg indospicine/kg BW/day for 32 days. The camels were sourced from the feral population in central Australia and purchased from a commercial supplier. Animal protocols for this study were approved by the Animal Ethics Committee of University of Queensland, Queensland, Australia (AEC Approval Number: SAFS/047/14/SLAI). The *Indigofera* plant material was fed daily in two equal meals at 9:00 am and 2:00 pm. On day 33, three of the animals (camels 1–3) were euthanized and tissues from 6 organs (muscle, heart, spleen, pancreas, liver, and kidney) were collected during necropsy and frozen (−20 °C) until needed. 

To exclude previous exposure to indospicine (or metabolites), the plasma collection started 10 days before the experimental feeding phase. Venous blood samples were collected from the jugular vein from all six animals (camels 1–6) in weekly intervals during the treatment phase. This was continued with the remaining three animals (camels 4–6) in weekly intervals until day 76, and then in fortnightly intervals until the end of the experiment (day 132), 100 days after cessation of the *Indigofera* feeding. Blood was collected in lithium heparin containers and spun for 10 min at 4400 rpm at 19 °C (Sigma 4K10, Osterode am Harz, Germany), after which the plasma was collected and frozen at −30 °C until analysis.

### 4.3. Indospicine Extraction

The tissue samples were extracted as previously described [26]. Camel organ tissues were thawed; 0.5 g was mixed with 25 mL 0.1% HFBA and homogenized for 15 s using a Polytron T25 homogenizer (Labtek, Brendale, Australia). The homogenates were cooled for 20 min at 4 °C before centrifugation (4500 rpm, 10 min, 18 °C). One mL of the supernatant was spiked with the internal standard, and an aliquot of 450 µL was transferred into a pre-rinsed Amicon Ultra, 0.5 mL, 3 K centrifugal filter (Merck, Millipore, Kilsyth, VIC, Australia), which was centrifuged at 10,000 rpm for 20 min. The filtrate was transferred into an autosampler vial for UHPLC-MS/MS analysis.

Plasma samples were thawed and diluted 25 times with 0.1% HFBA. An aliquot of the diluted plasma sample was spiked with the internal standard, and 450 μL were transferred to a pre-rinsed Amicon Ultra centrifugal filter. The filtrate was transferred into an autosampler vial for UHPLC-MS/MS analysis.

### 4.4. UHPLC-MS/MS Analysis

Quantification of the compounds was done according to a previously validated UHPLC-MS/MS method, with modifications [26]. Separation and quantification were carried out on a Shimadzu Nexera X2 UHPLC system (Shimadzu, Rydalmere, NSW, Australia), combined with a Shimadzu LCMS-8050 triple quadrupole mass spectrometer, equipped with an electrospray ionization (ESI) source operated in positive mode. Indospicine, 2-aminopimelamic acid, and 2-aminopimelic acid were separated at 35 °C on an Acquity BEH C18 column (Waters, Rydalmere, NSW, Australia) (100 mm × 2.1 mm id, 1.7 µm) with 0.1% HFBA and 100% acetonitrile as mobile phases A and B, respectively. The flow rate was set to 0.3 mL/min and the injection volume was 2 µL. The following gradient was applied: 1–70% B (3 min), 70% B (isocratic for 1 min), 70–100% B (0.5 min), the total run time was 6 min. The interface temperature was set to 300 °C, and the heating block to 400 °C. Nitrogen was used as nebulizing gas (2.0 L/min) and drying gas (5.0 L/min), compressed air was used as heating gas (10.0 L/min), and argon was used as the CID gas maintained at 270 kPa.

Indospicine was quantified using stable isotope dilution assay with two MS/MS transitions for each compound, *m/z* 174.2 → 84.0 and *m/z* 174.2 → 111.1 for indospicine, and *m/z* 177.2 → 113.0 and *m/z* 177.2 → 114.1 for the internal standard D_3_-l-indospicine.

External calibration curves were used for the quantification of 2-aminopimelamic acid and 2-aminopimelic acid. Specific SRMs were used for the identification: transition of *m/z* 175.00 → 112.05 (verified by transition of *m/z* 175.00 → 67.05) for 2-aminopimelamic acid, and transition of *m/z* 175.90 → 112.20 (verified by transition of *m/z* 175.90 → 69.15) for 2-aminopimelic acid. Unit resolution was used for both precursor and product *m/z* values. The collision energies (shown in Table 1) were optimized for each transition for maximum sensitivity.

## Figures and Tables

**Table 1:** Collision energy for quantifier and verifying single reaction monitoring (SRM) transitions for compounds **1**–**4**.

Compound	Collison Energy (eV)	
	Quantifier SRM	Verifier SRM
Indospicine (**1**)	−23.0	−17.0
D_3_-l-indospicine (**4**)	−17.0	−16.5
2-aminopimelamic acid (**2**)	−15.0	−28.0
2-aminopimelic acid (**3**)	−15.0	−20.0

## References

[1] Tan E.T.T., Materne C.M., Silcock R.G., D’Arcy B.R., Al Jassim R., Fletcher M.T. (2016). Seasonal and species variation of the hepatotoxin indospicine in Australian *Indigofera* legumes as measured by UPLC-MS/MS. J. Agric. Food. Chem..

[2] Wilson P.G., Rowe R. (2004). A revision of the Indigofereae (Fabaceae) in Australia. 1. *Indigastrum* and the simple or unifoliolate species of *Indigofera*. Telopea.

[3] Wilson P.G., Rowe R. (2008). A revision of the *Indigofereae* (Fabaceae) in Australia. 2. *Indigofera* species with trifoliolate and alternately pinnate leaves. Telopea.

[4] Wilson P.G., Rowe R. (2008). Three new species of *Indigofera* (Fabaceae: Faboideae) from Cape York Peninsula. Telopea.

[5] FitzGerald L.M., Fletcher M.T., Paul A.E.H., Mansfield C.S., O’Hara A.J. (2011). Hepatotoxicosis in dogs consuming a diet of camel meat contaminated with indospicine. Aust. Vet. J..

[6] Fletcher M.T., Al Jassim R.A.M., Cawtdell-Smith A.J. (2015). The occurrence and toxicity of indospicine to grazing animals. Agriculture.

[7] Hegarty M.P., Kelly W.R., McEwan D., Williams O.J., Cameron R. (1988). Hepatotoxicity to dogs of horse meat contaminated with indospicine. Aust. Vet. J..

[8] Hutton E.M., Windrum G.M., Kratzing C.C. (1958). Studies on the toxicity of *Indigofera endecaphylla*: I. Toxicity for rabbits. J. Nutr..

[9] Nordfeldt S., Henke L.A., Morita K., Matsumoto H., Takahash M., Younge O.R., Willers E.H., Cross R.F. (1952). Feeding tests with *Indigofera endecaphylla* Jacq. (Creeping indigo) and some observations on its poisonous effects on domestic animals. Hawaii Agric. Exp. Station Coll. Agric. Univ. Hawaii Tech. Bull..

[10] Hegarty M.P. (1986). Toxic amino acids in foods of animals and man. Proc. Nutr. Soc. Australia.

[11] Fowler M.E. (1983). Plant poisoning in free-living wild animals: A review. J. Wildl. Dis..

[12] Tan E.T.T., Al Jassim R., D’Arcy B.R., Fletcher M.T. (2016). Level of natural hepatotoxin (Indospicine) contamination in Australian camel meat. Food Addit. Contam. Part A.

[13] Tan E.T.T., Al Jassim R., Cawdell-Smith A.J., Ossedryver S.M., D’Arcy B.R., Fletcher M.T. (2016). Accumulation, persistence, and effects of indospicine residues in camels fed *Indigofera* plant. J. Agric. Food. Chem..

[14] Tan E.T.T., Al Jassim R., D’Arcy B.R., Fletcher M.T. (2017). In vitro biodegradation of hepatotoxic indospicine in *Indigofera spicata* and its degradation derivatives by camel foregut and cattle rumen fluids. J. Agric. Food. Chem..

[15] Sultan S., Giles C., Netzel G., Osborne S.A., Netzel M.E., Fletcher M.T. (2018). Release of indospicine from contaminated camel meat following cooking and simulated gastrointestinal digestion: Implications for human consumption. Toxins.

[16] Tan E.T.T., Yong K.W.L., Wong S.H., D’Arcy B.R., Al Jassim R., De Voss J.J., Fletcher M.T. (2016). Thermo-alkaline treatment as a practical degradation strategy to reduce indospicine contamination in camel meat. J. Agric. Food. Chem..

[17] Hegarty M.P., Pound A.W. (1970). Indospicine, a hepatotoxic amino acid from *Indigofera spicata*: Isolation, structure, and biological studies. Aust. J. Biol. Sci.

[18] Fletcher M.T., Reichmann K.G., Ossedryver S.M., McKenzie R.A., Carter P.D., Blaney B.J. (2018). Accumulation and depletion of indospicine in calves (*Bos taurus*) fed creeping indigo (*Indigofera spicata*). Anim. Prod. Sci..

[19] Madsen N.P., Hegarty M.P. (1970). Inhibition of rat liver homogenate arginase activity *in vitro* by the hepatotoxic amino acid indospicine. Biochem. Pharmacol..

[20] Biczó G., Hegyi P., Berczi S., Dósa S., Hracskó Z., Varga I.S., Iványi B., Venglovecz V., Wittmann T., Takács T. (2010). Inhibition of arginase activity ameliorates L-arginine-induced acute pancreatitis in rats. Pancreas.

[21] Emmanuel B. (1980). Urea cycle enzymes in tissues (liver, rumen epithelium, heart, kidney, lung and spleen) of sheep (*Ovis aries*). Comp. Biochem. Physiol. B Biochem. Mol. Biol..

[22] Morris S.M. (1992). Regulation of enzymes of urea and arginine synthesis. Annu. Rev. Nutr..

[23] Hegarty M.P., Court R.D. (1976). Indigofera spicata. Tropical Crops and Pastures Division of CSIRO 1975-1976 Annual Report.

[24] Sultan S., Osborne S.A., Addepalli R., Netzel G., Netzel M.E., Fletcher M.T. (2018). Indospicine cytotoxicity and transport in human cell lines. Food Chem..

[25] Lang C.S., Wong S.H., Chow S., Challinor V.L., Yong K.W.L., Fletcher M., Arthur D.M., Ng J.C., De Voss J.J. (2016). Synthesis of L-indospicine, [5,5,6-^2^H_3_]-l-indospicine and L-norindospicine. Org. Biomol. Chem..

[26] Tan E.T., Fletcher M.T., Yong K.W., D’Arcy B.R., Al Jassim R. (2014). Determination of hepatotoxic indospicine in Australian camel meat by ultra-performance liquid chromatography-tandem mass spectrometry. J. Agric. Food. Chem..

